# Temporal Changes in Breast Milk Fatty Acids Contents: A Case Study of Malay Breastfeeding Women

**DOI:** 10.3390/nu13010101

**Published:** 2020-12-30

**Authors:** Geok Lin Khor, Seok Shin Tan, Eline Stoutjesdijk, Kock Wai Tony Ng, Ilse Khouw, Marjolijn Bragt, Anne Schaafsma, D. A. Janneke Dijck-Brouwer, Frits A. J. Muskiet

**Affiliations:** 1 Department of Nutrition & Dietetics, School of Health Sciences, International Medical University, 57000 Kuala Lumpur, Malaysia; seokshin83@gmail.com (S.S.T.); drtonyngkw@gmail.com (K.W.T.N.); 2 Laboratory Medicine, University Medical Center Groningen and University of Groningen, 9713 GZ Groningen, The Netherlands; e.stoutjesdijk@umcg.nl (E.S.); d.a.j.dijck@umcg.nl (D.A.J.D.-B.); f.a.j.muskiet@umcg.nl (F.A.J.M.); 3 FrieslandCampina, 3800 BN Amersfoort, The Netherlands; Ilse.Tan-Khouw@frieslandcampina.com (I.K.); Marjolijn.Bragt@frieslandcampina.com (M.B.); Anne.Schaafsma@frieslandcampina.com (A.S.)

**Keywords:** breast milk fatty acids, temporal changes, maternal dietary intake, postpartum

## Abstract

The composition of human breast milk changes in the first two months of life, adapting itself to the evolving needs of the growing new-born. Lipids in milk are a source of energy, essential fatty acids (FA), fat-soluble vitamins, and vital bioactive components. Information on breast milk FA of Malaysian lactating women is scarce. Based on convenience sampling, a total of 20 Malay breastfeeding women who fulfilled the inclusion criteria were recruited. Breast milk was collected three times from each subject at consecutive intervals of 2–3 weeks apart. A total of 60 breast milk samples were collected and classified into “transitional milk” (*n* = 8), “early milk” (*n* = 26) and “mature milk” (*n* = 26). All milk samples were air freighted to University of Groningen, Netherlands for analysis. The dominant breast milk FA were oleic acid, constituting 33% of total fatty acids, followed by palmitic acid (26%). Both these FA and the essential FA, linoleic acid (10%) and alpha-linolenic acid (0.4%), showed no significant changes from transitional to mature milk. Breast milk ratio of n-6:n-3 polyunsaturated fatty acids (PUFA) was comparatively high, exceeding 10 throughout the lactation period, suggesting a healthier balance of PUFA intake is needed in pregnancy and at postpartum.

## 1. Introduction

Fats are important constituents of human breast milk providing energy and nutrients to support the growth and development of the breast-fed infant. In general, human breast milk fat content ranges from 3.5% to 4.5% during lactation with triglycerides as the main lipid fraction, accounting for about 95% of total lipids. Approximately, half of milk fatty acids are saturated fatty acids, with palmitic acid (C16:0) making up about 23% of the total fatty acids [1]. Overall, oleic acid (C18:1 n-9) constitutes the highest percentage (about 36%) of the total fatty acids in human milk.

The composition of human breast milk changes in the first two months of life, adapting itself to the evolving needs of the growing new-born. The first fluid that is expressed by the mother after delivery is colostrum, which has high immunologic components and low concentrations of lactose, indicating its primary functions to be immunologic rather than nutritional [2]. In this respect, Gila-Diaz et al. (2019) [3] have provided an insightful elaboration on the presence of several bioactive compounds in breast milk, which contribute to the maturation of the immune system of the breastfed infant, among other important aspects. At the beginning of lactation, milk fat also contains more capric acid (C10:0) and lauric acid (C12:0) as these are readily metabolised and are also believed to confer immunological benefits.

As lactation proceeds, lactose concentration increases indicating secretory activation and the production of transitional milk [2]. Transitional milk, typically occurring from day 7 to day 10 postpartum to 2 weeks postpartum, contains increasingly higher concentrations of energy and nutrients to support the nutritional and developmental needs of the rapidly growing infant. By four to six weeks postpartum, human milk is considered fully mature.

As fat is recognised as a highly variable component in breastmilk, and its fatty acid composition is known to be influenced by multiple external factors including maternal diet, the relationship between breast milk fatty acids and maternal dietary intake has been extensively studied. In a systematic review of the impact of maternal nutrition on breast milk composition, which included 17 studies on dietary effects on breast milk fatty acids, Bravi et al. (2016) [4] reported several limitations in comparing the studies. These included heterogeneity in study designs and lack of control for confounding factors. In their meta-analysis of 41 studies, Gidrewicz and Fenton (2014) [5] revealed more similarities than differences between preterm and term milk for energy, fat, oligosaccharides, calcium, and phosphorus.

The exclusively breastfed infant depends on human milk for essential fatty acids namely, linoleic acid (LA) (18:2 n-6) and alpha-linolenic acid (ALA) (C18:3 n-3). Both LA and ALA serve as precursors to physiologically important polyunsaturated metabolites, including arachidonic acid (AA) (20:4 n-6), eicosapentaenoic acid (EPA) (20:5 n-3) and docosahexaenoic acid (DHA) (22:6 n-3). The role of DHA in infant nutrition is of particular importance because it is accumulated during perinatal development specifically in the membrane lipids of the brain and retina, where DHA plays vital roles in visual acuity vision and motor systems in newborns [6].

EPA and DHA are derived mainly from fish and other seafoods including seaweed [7]. Several studies world-wide have reported low amounts of EPA and DHA in maternal diets [8,9,10]. Low maternal intake of DHA in particular may lead to low breast milk DHA levels, which in turn place the infant at risk of dysfunction of the central nervous system, plausibly impairing cognition and vision [11].

Given the important roles of fatty acids in breast milk for infant growth and development, and in view of a lack of data on breast milk fatty acids in Malaysia, this study is aimed at generating information on fatty acids composition and maternal dietary intake among Malay lactating women. Such information is also pertinent in relation to the notable increase in the prevalence of breastfeeding among Malaysian women in recent years. The national prevalence of exclusive breastfeeding increased from 19.3% in 2006 to 47.1% in 2016 according to the National Health and Morbidity Survey (NHMS) of 2006 and 2016 [12]. The NHMS also showed that exclusive breastfeeding prevalence in urban areas has risen significantly over the same period from 12.9% to 48.3%.

We have lately published a case study on the breastmilk mineral contents of Malay lactating women living in urban areas [13]. The present report follows up on the fatty acids contents of breast milk samples that were collected from the Malay lactating women.

## 2. Methodology

### 2.1. Ethics Approval and Consent to Participate

This study was undertaken in agreement with the Helsinki declaration of 1975 as revised in 2013. The International Medical University Joint Committee on Research and Ethics gave approval for this study with the approval number of IMU R123. The International Medical University Joint Committee on Research and Ethics operates in accordance with the International Conference of Harmonization/Good Clinical Practice Guidelines (ICH-GCP) Guidelines, the Declaration of Helsinki 2000, and Malaysian Good Clinical Practice. The study proposal was also registered in the Netherlands National Trial Register with the registration number of NTR 4404.

### 2.2. Study Design and Subjects

This is a cross-sectional study conducted in an urban residential area of Kuala Lumpur, the capital city of Malaysia. The study location was purposively selected for its largely Malay residents. Malay women were selected as they are known to breastfeed longer on average than other ethnic urban populations [14]. In addition, Malay women are known to have culturally different dietary practices during pregnancy and postpartum that might affect the milk contents [15,16].

### 2.3. Inclusion and Exclusion Criteria

#### 2.3.1. Inclusion Criteria

1. Apparently healthy Malay women aged 20–40 years
2. Women breastfeeding throughout the milk collection period
3. Infant born singleton, gestational age ≥ 37 weeks, birth weight ≥ 2500 g

#### 2.3.2. Exclusion Criteria

1. Infant at recruitment older than 2 weeks to exclude colostrum milk feeding
2. Breastfeeding women on special diets
3. Breastfeeding women taking dietary supplements including fatty acid supplements
4. Breastfeeding women fasting during milk collection period
5. Breastfeeding women or child feeling sick at recruitment

### 2.4. Subject Recruitment Process

Recruitment of subjects was based on community feedback on households with breastfeeding women. The study protocol was explained to women who fulfilled the inclusion criteria. However, several did not agree to participate, giving reasons such as “too busy to commit”, “husband did not agree” and “working outside the home”. Through the ‘snowball’ approach, a total of 20 women who fulfilled the study criteria agreed to participate in the study. All gave written consent for their participation. Appointments were scheduled for three consecutive collections of breast milk samples and interviewing regarding the dietary intake of the women.

### 2.5. Collection of Milk Samples

Throughout the milk collection period, milk samples were collected in the morning to standardise the timing of collection and to minimise circadian effects on milk composition [17]. Before collecting milk sample, the nipples and areolas of the breast were cleaned with 70% alcohol ethyl alcohol. Complete milk was expressed from one breast by a trained research assistant using a manual breast pump (Phillips Avent model SCF 330-13). In this way, at least 25 mL breast milk from each subject was collected and transferred into a sterile tube. The milk samples collected were placed together with ice packs inside a portable insulated container and transported within 5-h of collection to the institution laboratory (IMU) for storage at minus 80 °C. Overall, breast milk was collected three times from each subject at consecutive intervals of 2–3 weeks apart. Hence a total of 60 breast milk samples were collected from the 20 subjects.

### 2.6. Determination of Breast Milk Fatty Acids

The milk samples were air freighted on dry ice to the University Medical Center Groningen (UMCG), The Netherlands for fatty acids composition analysis. They were stored at minus 20 °C until further analysis. The breast milk samples were thawed at room temperature prior to analysis. An aliquot of 100 uL was transferred into a Telfon-sealable tube (Sovirel), containing 2 mL of methanol: 6 mol/L HCl (5:1, *v*/*v*), 1 mg of butylated hydroxytoluene (antioxidant), 100 uL of 5:0–15:0 and 50 uL of 17:0 internal quantification standards. These ready-to-transmethylate fatty acid mixtures were trans-methylated by heating to 90 °C for 4-h. After extraction, analysis of fatty acids methyl esters (FAME) was performed by capillary gas chromatography/ flame ionization detection using a series of odd-chain numbered fatty acids (5:0–17:0) as internal quantification standards. In brief, the samples were injected into gas chromatography (Hewlett Packard, Model 5880), equipped with a 25 m × 0.25 mm I.D. CP-Sil-88-coated (film thickness 0.25 um) fused-silica column (Chrompack, Middelburg, The Netherlands). The flow rate for helium gas was set at 0.65 mL/min, split ratio of 1:15, temperature for flame ionization detector of 300 °C and injector temperature of 200 °C. The oven temperature programme was set as 150 °C, 1 °C/min to 200 °C, 10 min at 200 °C [18,19]. The composition of fatty acids in the breast milk samples was recorded as percentage of total fatty acids (g%).

Based on the age of the infants when the breast milk was collected, this study categorised lactation stage according to the terminology used in Ballard and Morrow (2013) [2] and Li et al. (2016) [20]. Milk samples collected at week 2 to week 3 postpartum were labelled as “transitional” milk (T1), at week >3 to week 8 as “early” milk (T2), and at week >8 to week 16 as “mature” milk (T3). Out of the total 60 breast milk samples collected, there were 8 samples of transitional breast milk, 26 samples of early milk and 26 samples of mature milk.

### 2.7. Maternal Dietary Intake Assessment

During each milk collection, the breastfeeding women were interviewed on their dietary intake using the 24-h recall method. In this way, a total of three 24-h recall records of food intake were obtained from each subject at consecutive 2–3 week intervals. The macronutrients and fatty acid contents of various food consumed by the subjects were computed using the Malaysian Food Composition [21], United States Department of Agriculture (USDA) Nutrient Database [22] and the Australia Food, Supplement and Nutrient Database (AUSNUT) [23] in the above order of priority. The results were reported as mean ± SD for macronutrients, saturated fatty acids (SFA), monounsaturated fatty acids (MUFA) and polyunsaturated fatty acids (PUFA) and as percentage meeting the daily recommendations for Malaysia [24].

### 2.8. Statistical Analysis

Statistical analyses were conducted using the SPSS (Statistical Package for Social Sciences) version 20. Descriptive statistics were expressed as mean ± standard deviation (SD). The breast milk fatty acid contents at different lactation stages as well as the maternal dietary intake of fatty acids at the three collection times were analysed by ANOVA and post hoc Fisher’s Least Significant Differences (LSD) tests. Significance level was set at *p* < 0.05 for all analyses.

## 3. Results

### 3.1. Socio-Demographic Background

The socio-demographic background of the breastfeeding women was previously described in Tan et al. (2020) [13]. Briefly, the women and their spouses had a mean age of 31.4 ± 6.1 and 33.8 ± 7.4 years, respectively. The majority of the women and their spouses had completed more than 10 years of formal schooling. While the majority of the women were housewives, their spouses were employed in the service and production industry.

The women had a mean body mass index (BMI) of 26.0 ± 5.1 kg/m^2^, indicating that the subjects had normal to obese BMI status. The authors considered the small sample size (*n* = 20) in this study precludes meaningful statistical determination of the association between maternal BMI and breast milk contents. While positive associations between maternal BMI and breast milk fat contents have been reported [25,26], the latter has also pointed out contradictory findings.

Among the infants in the study, there were more female (70%) than male. Data on their birth weight, ranging from 2840 g to 3780 g, indicates that none of the infants were born with low birth weight.

### 3.2. Breast Milk Fatty Acid Concentrations

Oleic acid (C18:1 n-9) is the predominant fatty acid in the breast milk constituting about 33% of the total fatty acids at each of the three stages of lactation (T1, T2 & T3) (Table 1). The next highest level is palmitic acid (C16:0) forming about 26% followed by linoleic acid (C18:2 n-6) (approximately 10%) and lauric acid (C12:0) (about 9%). These relative proportions of the breast milk fatty acids do not change significantly as lactation progresses from the transitional milk to mature milk stage.

It is also noted that the concentrations of the essential fatty acids, i.e., linoleic acid (18:2 n-6) and alpha-linolenic acid (18:3 n-3), which formed about 10% and 0.4% respectively of the total fatty acids, showed no significant changes across the lactation stages.

The total concentration of saturated fatty acids (SFA) increases somewhat from 49.4 ± 1.55% in the transitional milk to 50.8 ± 0.78% in mature milk (Table 1). The dominant SFA is palmitic acid (C16:0) followed by lauric acid (C12:0), myristic acid (C14:0) and stearic acid (C18:0). Meanwhile, the sum of monounsaturated fatty acids (MUFA) decreases significantly from 38.2 ± 1.13% to 37.1 ± 0.61% as lactation progresses. Total polyunsaturated fatty acids (PUFA) concentration remains almost constant at about 12% throughout the lactation stages. Out of the total PUFA content, n-3 PUFA constitutes only about 1% of the total fatty acids across the stages of lactation. The n-3 PUFA in breast milk are α-linolenic acid (C18:3), eicosapentaenoic acid (EPA) (C20:5) and docosahexaenoic acid (DHA) (C22:6). In contrast, the sum of n-6 PUFA forms about 11% of the total PUFA, resulting in the breast milk ratio of n-6 to n-3 PUFA in this study at approximately 10 to 11 through the lactation period.

### 3.3. Maternal Dietary Intake

Dietary intake of the breastfeeding women is shown according to the three-time stages of lactation, (T1, T2 and T3). Maternal intake is also shown as percentage of meeting Malaysia’s daily recommended intake (RNI) for energy, macronutrients, SFA, MUFA and PUFA [24]. Overall, the average daily intake of total energy and protein of the breastfeeding women exceeded the respective RNI recommendation of 2400 kilocalories and 71 g (Table 2). The mean daily energy intake increased significantly from 2270.9 ± 1004.8 kcals at the transitional milk stage (T1) to 2435.9 ± 806.1 kcals at mature milk stage (T3). Likewise, the daily intake of protein increased significantly from 83.6 ± 37.9 g at T1 to 94.3 ± 39.7 g at T3. While rice and noodles constituted the primary sources of calories among the women postpartum, their chief protein sources were meat (beef, chicken), fish especially “ikan kembong” (Rastrelliger kanagurta), milk, eggs and soybean products (soya milk, soya curd).

In general, the total fat intake of the breastfeeding women approaches 90.3–108.0% of the Malaysian RNI level for moderately active lactating women. This intake level did not change significantly across the lactation stages. Out of the total fat intake, the mean intake of SFA of 26.7 g per day was about 60% of the RNI level for SFA. As a percentage of total fatty acids, the intake of SFA showed a significant declining trend from 25.1% at T1 to 21.5% at T3. In general, postpartum maternal intake levels of MUFA and PUFA were below the RNI levels, at respectively 36.5–45.6% and 45.2–70.2% across the three stages of lactation.

The main sources of fat consumed by the breastfeeding women were cooking oil, coconut cream (“santan”), fish and meat. Out of these, meat, palm oil and coconut cream constituted the major sources of SFA. Palm oil is a popular cooking oil in Malaysia, containing approximately 50% SFA, with 44% palmitic acid (C16:0). Palm oil is also a dietary source of unsaturated fatty acids, as it comprises 40% oleic acid (C18:1n−9) and 10% linoleic acid (C18:2n−6) and linolenic acid (C18:3n−3) [27]. Aside from palm oil, other popular types of cooking oil in Malaysia include sunflower oil, soybean oil, peanut oil, corn oil and mixed blend of vegetable oils. These oils contribute to n-6 PUFA intake of the lactating women. Intake of n-3 PUFA is likely to be derived from a wide variety of fish that the women reported consuming, including mackerel, sardine, tuna, red snapper, anchovy, pomfret and catfish.

Vegetables were consumed daily whereas legumes and nuts were taken on a weekly basis. Green leafy vegetables that were popular among the breastfeeding women include “sawi” (mustard; Brassica sp), “kangkong” (Ipomoea aquatica) and spinach (Amaranthaceae), as the women believed that taking vegetables such as mustard and kangkong frequently would increase production of breast milk. In general, the daily intake of the breastfeeding women showed wide inter-subject variations at each point of assessment, as indicated by the standard deviation range for the mean values.

## 4. Discussion

Human milk has been traditionally recommended as the ideal source of energy, nutrients and bioactive components to support the healthy growth and development of infants [28,29]. As fat is the most variable component in breastmilk and its composition is known to vary with external factors including maternal diet, the relationship between breast milk fatty acids and maternal dietary intake has been extensively studied. In a review of 14 studies from 9 European countries and 10 studies from 7 African countries on fatty acids in mature human milk, Koletzko et al. (1992) [30] reported that “diets in different geographic locations seem to have little effect on the milk content of 20 and 22 carbon long-chain polyunsaturated fatty acids (LCP), with the exception of relatively high n-3 fatty acids in the milk of African women consuming a large proportion of dietary fat from sea fish”. The authors concluded that “the milk secretion of n-6 LCP does not appear to depend on maternal dietary intake of preformed LCP. Metabolic processes appear to be important in regulating human milk LCP”. A somewhat similar finding was concluded by Yuhas et al. (2006) [31], who, in examining breast milk fatty acids of women from Asia, Australia and several western countries, stated that “the proportion of saturated and monounsaturated FA are relatively constant across a large number of countries, whereas the level of some of the PUFA, especially DHA, are highly variable”. In an analysis of pooled data from 55 countries world-wide, Floris et al. (2020) [32] showed that a number of saturated fatty acids, chief of which were palmitic acid and the essential fatty acids, remain relatively stable throughout the lactation stages from colostrum to mature milk. The authors also reported that “almost all n-6 PUFA, including AA and DHA decrease steadily over the lactational stages, while the n-3 PUFA including ALA and EPA were relatively stable at all time points”.

Against the background of those reviews, we compared the major fatty acids contents in the mature milk of this study with those reported by other countries (Table 3). For total saturated fatty acids (SFA), the highest level is in the breast milk from Malaysia at 49.1% (of total fatty acids) followed by Switzerland, South Korea, Poland and Canada, each within approximately 40–44%. This seemingly narrow range of total SFA in mature breast milk from diverse Asian and European countries confirmed the reviews of others that the proportion of breast milk SFA is relatively constant across different populations. Another common characteristic about milk SFA of different countries is the predominance of palmitic acid (C16:0), which constitutes approximately half of the total SFA content in each country. A known unique feature of breast milk SFA is that a major proportion (70%) of the palmitic acid is esterified in the middle position (sn-2 position) of triacylglycerols. This facilitates pancreatic lipolysis yielding a palmitoyl-monoglycerol, that is water soluble and well absorbed, hence enabling efficient digestion and absorption of C16:0 and calcium [29,33].

To account for the exceptional SFA features in human milk, German and Dillard (2010) [38] expounded that, in the context of evolution, milk SFA has developed through natural selection to support growth and survival of mammalian offspring. These biological roles include having the short-chain SFA, butyric acid (C4:0) being preferentially hydrolysed and absorbed, thus serving as a ready source of energy, while caproic acid (C6:0) to capric acid (C10:0) are shown to exhibit antimicrobial activities. The significant presence of the triacylglycerol backbone structure of milk palmitic acid at the sn-2 position is suggested to have a positive impact on lipoprotein metabolism in infants [39], and also to impart benefits for neonatal intestinal and immunological health outcomes [40].

Similar to the fact that total SFA content is rather constant in mature breast milk from different populations, total MUFA is also reported to occur within a narrow range of concentrations in mature milk [29,31]. Among the Asian and European countries under consideration here (Table 3), total MUFA content lies between 36–44%, with the Malaysian case at 39%. Oleic acid (18:1 n-9) is the most abundant MUFA among the different populations, similar to other findings world-wide. While oleic acid has been shown to possess an immunomodulatory function, its role in immune responses remains inconclusive [40].

The total n-6 PUFA content in breast milk ranks highest in China (24.1%) followed by Malaysia (11.2%) (Table 3). Linoleic acid is the predominant n-6 fatty acid in all countries followed distantly by arachidonic acid (C20:4 n-6) e.g., 10% and 0.39% respectively in the case of Malaysia. The dominant dietary source for the high breast milk n-6 PUFA for China is cooking oils, chiefly soybean oil and peanut oil [9], whilst that for Malaysia is attributed to palm oil due primarily to its availability, affordability and good frying quality.

As for the total n-3 PUFA content, it constitutes a relatively small percentage of the overall total fatty acids in breast milk, e.g., ranging from 1.05% for Malaysia to 3.44% for Bolivia. The main n-3 fatty acids are α-linolenic acid (C18:3 n-3) and DHA (C22:6 n-3) followed by EPA (C20:5 n-3). The amount of DHA in breast milk ranges from 0.18% (Canada) to 0.7% (Poland), with 0.49% for Malaysia. The DHA concentration for Malaysia appears adequate for breast fed infants based on the proposition of Koletzko (2016) [29], that “breastfeeding women need to achieve a daily DHA intake of at least 200 mg to provide milk with a DHA content of at least 0.3%, which is required for a fully breastfed infant to obtain the daily supply of about 100 mg DHA/day considered desirable to meet metabolic needs”.

The dietary source of DHA of the Malay lactating women is likely to be meat and fish. During the confinement period of about 30–60 days after delivery, Malay mothers are encouraged to consume more meat and fish as they are considered “hot” foods, which “can restore the internal heat lost from the body during delivery” [15]. The women are also encouraged to consume fish frequently postpartum, as it is a cultural belief that eating fish after delivery promotes milk flow. In this study, a wide variety of fish including anchovy, sardine and mackerel were reportedly consumed by the breastfeeding women.

In comparing the average n-6:n-3 PUFA ratio, China and Malaysia showed a comparatively high ratio of 12.5 and 10.1 respectively, while the ratio values for South Korea (6.7), Canada (6.5) and United States (7.6) were closer to the mean ratio of 6.53 ± 1.72 reported for 55 countries world-wide [32]. In a large multi-ethnic CHILD (Canadian Healthy Infant Longitudinal Development) cohort in Canada, Moossavi et al. (2019) [35] showed that mothers who consumed fatty cold water fish or took fish oil supplements during pregnancy and/or lactation had higher proportions of n-3 PUFAs and lower ratios of total n-6:n-3 PUFAs. The case of the relatively low n-6:n-3 ratio of 3.48 for Bolivia is explained by their subsistence on a plant-based diet with little intake of dietary energy from processed foods [37].

### Strengths and Limitations of Study

To the best of our knowledge, this is the only study that provides information on the changes in the composition of breast milk fatty acids of Malay breastfeeding women, from postpartum week 2–3 (translational milk stage), to week 3–8 (early milk) and to week 8–16 (mature milk). Maternal dietary intake was also assessed using the 24-h recall method at each of the three lactation stages when breast milk was collected. Nonetheless, owing to the small sample size and low statistical power, we did not determine the correlations between maternal intake and breast milk fatty acids content. Overall, the maternal intake data provided insightful quantitative and descriptive perspectives on the postpartum dietary intake practices of Malay breastfeeding women. A strength of this study is that the breast milk fatty acids concentrations were determined at the Laboratory Medicine of the University Medical Center Groningen and University of Groningen, the Netherlands, which adheres to internal and external quality assessment.

## 5. Conclusions

This study has provided some useful insights into the temporal changes of breast milk fatty acids of Malay breastfeeding women. The predominant breast milk fatty acids are oleic acid (C18:1 n-9) constituting about 33% of the total fatty acids, followed by palmitic acid (C16:0) (about 26%). Both these fatty acids did not change significantly in concentrations in the transitional milk through the early milk and in the mature milk. Likewise, the concentrations of the essential fatty acids namely, linoleic acid (18:2 n-6) (10%) and alpha-linolenic acid (18:3 n-3) (0.4%), showed no significant change throughout the lactation stages. Total polyunsaturated fatty acids (PUFA) concentration remains almost constant at about 12% as lactation progresses. Out of the total PUFA, n-3 PUFA constitutes only about 1% of the total fatty acids throughout lactation. The breast milk ratio of n-6 to n-3 PUFA of 10–11 during the lactation period is relatively high compared to the global mean ratio of approximately 6–7. In view of the physiological importance of n3 PUFA for infant growth and development, this finding indicates the need for advocating a healthier balance of PUFA intake in pregnancy and at postpartum among Malaysian women.

The above results should be verified in a larger sample size, taking into consideration that the fatty acid composition of breast milk is influenced by a myriad of factors, including current and long-term maternal diet, maternal nutritional status, diurnal variations in fatty acid synthesis, and variations between collection methods within lactation stage [41]. Also, in view of the growing prevalence of childhood obesity in Malaysia and increasing prevalence of exclusive breastfeeding practice, it is suggested that future breast milk composition studies include investigating the roles of bioactive components in breast milk [42].

## Figures and Tables

**Table 1:** Breast milk fatty acids concentrations according to the stage of lactation (T1, T2 & T3).

	Stage of Lactation		
Breast Milk Fatty Acids Contents % of Total Fatty Acids	Transitional Milk (T1) 2–3 Weeks (*n* = 8)	Early Milk (T2) >3–8 Weeks (*n* = 26)	Mature Milk (T3) >8–16 Weeks (*n* = 26)
	Mean ± SD		
6:0 (Caproic acid)	0.11 ± 0.02	0.12 ± 0.01	0.13 ± 0.01
8:0 (Caprylic acid)	0.42 ± 0.03	0.45 ± 0.03	0.42 ± 0.02
10:0 (Capric acid)	2.29 ± 0.23	2.13 ± 0.15	2.09 ± 0.07
12:0 (Lauric acid)	9.61 ± 0.84 ^a^	8.14 ± 0.77 ^b^	8.96 ± 0.46 ^a,b^
14:0 (Myristic acid)	7.22 ± 0.55 ^a^	6.20 ± 0.55 ^b^	7.42 ± 0.41 ^a^
14:1 *n*-5 (Myristoleic acid)	0.04 ± 0.01 ^a^	0.13 ± 0.01 ^b^	0.09 ± 0.01 ^a,b^
16:0 (Palmitic acid)	25.2 ± 1.25 ^a^	26.3 ± 0.35 ^b^	26.8 ± 0.47 ^b^
16:1 *n*-7 (Palmitoleic acid)	3.09 ± 0.38	3.26 ± 0.19	2.61 ± 0.17
18:0 (Stearic acid)	4.26 ± 0.04	4.81 ± 0.14	4.70 ± 0.13
18:1 *n*-7 (Vaccenic acid)	1.58 ± 0.26	1.85 ± 0.06	1.53 ± 0.09
18:1 n-9 (Oleic acid)	33.0 ± 1.11 ^a,b^	33.7 ± 0.81 ^a^	32.5 ± 0.47 ^b^
18:2 n-6 (Linoleic acid)	10.1 ± 0.60	10.1 ± 0.32	9.83 ± 0.26
18:3 n-3 (α-Linolenic acid)	0.44 ± 0.2	0.35 ± 0.02	0.32 ± 0.03
18:3 n-6 (γ-Linolenic acid)	0.09 ± 0.02	0.09 ± 0.01	0.08 ± 0.01
20:0 (Arachidic acid)	0.14 ± 0.01	0.15 ± 0.01	0.16 ± 0.01
20:1 *n*-9 (Eicosenoic acid)	0.31 ± 0.04	0.30 ± 0.01	0.29 ± 0.02
20:2 n-6 (Eicosadienoic acid)	0.20 ± 0.01	0.20 ± 0.01	0.19 ± 0.02
20:3 n-6 (Dihomo--linolenic acid)	0.33 ± 0.04	0.31 ± 0.02	0.30 ± 0.02
20:4 n-6 (Arachidonic acid) (ARA)	0.43 ± 0.04	0.38 ± 0.02	0.37 ± 0.02
20:5 n-3 (Eicosapentaenoic acid) (EPA)	0.05 ± 0.01	0.06 ± 0.01	0.07 ± 0.01
22:0 (Behenic acid)	0.07 ± 0.01	0.07 ± 0.01	0.08 ± 0.01
22:4 n-6 (Docosatetraenoic acid)	0.10 ± 0.01	0.08 ± 0.01	0.08 ± 0.01
22:5 n-3 (Docosapentaenoic acid)	0.11 ± 0.01	0.11 ± 0.01	0.13 ± 0.01
22:5 n-6 (Docosapentaenoic acid)	0.01 ± 0.01	0.06 ± 0.01	0.07 ± 0.01
22:6 n-3 (Docosahexaenoic acid) (DHA)	0.45 ± 0.06	0.47 ± 0.05	0.56 ± 0.05
24:0 (Lignoceric acid)	0.07 ± 0.01	0.07 ± 0.01	0.08 ± 0.01
24:1 *n*-9 (Nervonic acid)	0.07 ± 0.01	0.07 ± 0.01	0.07 ± 0.01
Sum SFA	49.4 ± 1.55 ^a^	48.5 ± 1.20 ^a^	50.8 ± 0.78 ^b^
Sum MUFA	38.2 ± 1.13 ^a^	39.3 ± 0.95 ^a^	37.1 ± 0.61 ^b^
Sum PUFA	12.4 ± 0.79	12.2 ± 0.32	12.0 ± 0.34
Sum n-3	1.06 ± 0.16	0.99 ± 0.06	1.09 ± 0.08
Sum *n*-5	0.04 ± 0.01 ^a^	0.13 ± 0.01 ^b^	0.09 ± 0.01 ^a,b^
Sum n-6	11.3 ± 0.65	11.2 ± 0.33	10.9 ± 0.29
Sum *n*-7	4.71 ± 0.47 ^a,b^	5.11 ± 0.24 ^a^	4.16 ± 0.24 ^b^
Sum *n*-9	33.5 ± 1.14 ^a,b^	34.1 ± 0.82 ^a^	32.9 ± 0.48 ^b^
n-6 to n-3 ratio	10.7 ± 3.92	11.3 ± 5.79	10.1 ± 3.77
ARA to DHA ratio	0.96 ± 0.66 ^a^	0.80 ± 0.34 ^a^	0.66 ± 0.41 ^b^

Mean values in the same row with different alphabets indicate significant difference (*p* < 0.05) based on ANOVA and post hoc LSD tests. Saturated Fatty Acids (SFA): 6:0 (Caproic acid), 8:0 (Caprylic acid), 10:0 (Capric acid), 12:0 (Lauric acid), 14:0 (Myristic acid), 16:0 (Palmitic acid), 18:0 (Stearic acid), 20:0 (Arachidic acid), 22:0 (Behenic acid), 24:0 (Lignoceric acid). Monounsaturated Fatty Acids (MUFA): 14:1 n-5 (Myristoleic acid), 16:1 n-7 (Palmitoleic acid), 18:1 n-7 (Vaccenic acid), 18:1 n-9 (Oleic acid), 20:1 n-9 (Eicosenoic acid), 24:1 n-9 (Nervonic acid). Polyunsaturated Fatty Acids (PUFA): 18:2 n-6 (Linoleic acid), 18:3 n-3 (α-Linolenic acid), 18:3 n-6 (γ-Linolenic acid), 20:2 n-6 (Eicosadienoic acid), 20:3 n-6 (Dihomo-linolenic acid), 20:4 n-6 (Arachidonic acid), 20:5 n-3 (Eicosapentaenoic acid), 22:4 n-6 (Docosatetraenoic acid), 22:5 n-3 (Docosapentaenoic acid), 22:5 n-6 (Docosapentaenoic acid), 22:6 n-3 (Docosahexaenoic acid).

**Table 2:** Maternal dietary intake at three consecutive assessments (T1, T2, T3) (Mean ± SD) and % meeting daily recommendation (* RNI).

Maternal Intake	T1 (*n* = 20)		T2 (*n* = 20)		T3 (*n* = 20)		Overall (*n* = 20)		* RNI for Lactation
	(Mean ± SD)	%RNI	(Mean ± SD)	%RNI	(Mean ± SD)	%RNI	(Mean ± SD)	%RNI	
Energy (kcal/day)	2270.9 ± 1004.8 ^a^	94.6 ± 41.9	2535.4 ± 983.1 ^b^	105.6 ± 41.0	2435.9 ± 806.1 ^b^	101.5 ± 33.6	2414.1 ± 931.3	100.6 ± 38.8	2400.0
Carbohydrate (g/day)	338.2 ± 151.6 ^a^	86.7–112.7 ± 38.9–50.5	368.2 ± 164.3 ^b^	94.4–122.7 ± 42.1–54.8	340.5 ± 127.8 ^a^	87.3–113.5 ± 32.8–42.6	348.9 ± 147.8	89.5–116.3 ± 37.9–49.3	300.0–390.0
Protein (g/day)	83.6 ± 37.9 ^a^	117.7 ± 53.4	100.8 ± 59.9 ^b^	141.9 ± 84.4	94.3 ± 39.7 ^b^	132.8 ± 55.9	92.9 ± 45.8	130.8 ± 64.5	71.0
Fat (g/day)	64.6 ± 38.9 ^a^	81.8–97.9 ± 49.2–58.9	72.2 ± 37.1 ^a,b^	91.4–109.4 ± 47.0–56.2	77.1 ± 29.3 ^b^	97.6–116.8 ± 37.1–44.4	71.3 ± 35.1	90.3–108.0 ± 44.4–53.2	66.0–79.0
Cholesterol (mg/day)	163.2 ± 123.6 ^a^	54.4 ± 41.2	412.7 ± 660.7 ^b^	137.6 ± 220.2	285.2 ± 195.6 ^c^	95.1 ± 65.2	287.1 ± 326.6	95.7 ± 108.9	300
Sum of Fatty Acids									
SFA (g)	16.2 ± 11.9	60.7 ± 44.6	17.3 ± 10.9	64.8 ± 40.8	16.6 ± 11.1	62.2 ± 41.6	16.7 ± 11.3	62.5 ± 42.3	26.7
MUFA (g)	12.6 ± 8.0 ^a^	31.5–39.4 ± 20.0–25.0	16.2 ± 10.2 ^b^	40.5–50.6 ± 25.5–31.9	15.0 ± 9.3 ^b^	37.5–46.9 ± 23.3–29.1	14.6 ± 9.2	36.5–45.6 ± 23.0–28.8	32.0–40.0
PUFA (g)	8.1 ± 7.1 ^a^	37.0–57.4 ± 32.4–50.4	11.2 ± 6.7 ^b^	51.1–79.4 ± 30.6–47.5	10.3 ± 6.5 ^b^	47.0–73.0 ± 29.7–46.1	9.9 ± 6.8	45.2–70.2 ± 31.1–48.2	14.1–21.9
SFA (%)	25.1 ^a^		24.0 ^a^		21.5 ^b^		23.4		
MUFA (%)	19.5 ^a^		22.4 ^b^		19.5 ^a^		20.5		
PUFA (%)	12.5 ^a^		15.5 ^b^		13.4 ^a,b^		13.9		

Maternal dietary intake assessed at the stage of lactation defined as transitional milk (T1), early milk (T2) and established milk (T3). Maternal intake determined using 24-h recall; mean intake is derived from 3 times 24-h recall for each subject. Mean values in the same row with different alphabets indicate significant difference (*p* < 0.05) based on one-way ANOVA and post hoc (LSD). * RNI: Recommended Nutrient Intakes for Malaysia [24].

**Table 3:** Comparison of fatty acids contents (% of total fatty acids) in mature breast milk (>2 weeks and ≤16 weeks postpartum) from selected countries.

	Malaysia Mean ± SD	China Mean ± SD	South Korea Mean ± SD	Canada Mean ± SD	Switzerland Median (IQR)	Poland Mean ± SD	* Bolivia Mean ± SD/Median (IQR)	* USA Mean ± SD/Median (IQR)
C14:0 myristic acid	7.42 ± 0.41	4.2 ± 1.7		5.97 ± 1.80	6.27 (1.93)	9.5 ± 2.5	9.81 ± 4.18	8.67 ± 2.81
C16:0 palmitic acid	26.8 ± 0.47	19.8 ± 2.6		20.90 ± 2.76	23.29 (3.31)	19.7 ± 2.5	24.96 ± 3.13	20.00 ± 2.64
C18:0 stearic acid	4.70 ± 0.13	5.1 ± 1.1		6.54 ± 1.31	6.75 (1.69)	6.4 ± 1.5	5.54 ± 1.49	6.67 ± 1.51
Total SFA	49.1 ± 4.9	36.2 ± 4.7	42.1 ± 5.6	39.75 ± 5.00	43.86 (5.93)	41.9 ± 4.9		
Oleic Acid C18:1 n-9	32.5 ± 0.47	31.9 ± 3.6		37.05 ± 3.59	37.67 (4.82)	35.4 ± 3.1		
Total MUFA	38.5 ± 3.8	36.9 ± 4.1	36.3 ± 4.9	43.06 ± 3.59	43.84 (4.96)	39.6 ± 3.1		
Total PUFA	12.3 ± 1.8		21.5 ± 4.7		11.77 (3.43)	15.1 ± 3.4		
Total n-3 PUFA	1.05 ± 0.10	1.9 ± 0.9	3.01 ± 1.3	2.39 ± 0.70	1.11 (0.36)	2.7 ± 0.9	3.44	2.00
α-Linolenic Acid C18:3	0.37 ± 0.08	1.5 ± 0.9		1.92 ± 0.61	0.74 (0.30)	1.5 ± 0.6	1.64 (0.60–2.68)	1.39 (0.61–2.17)
EPA C20:5	0.06 ± 0.01	0.05 ± 0.07	0.15 ± 0.12	0.08 ± 0.05	0.06 (0.04)	0.2 ± 0.1	1.17 (0.04–0.30)	0.06 (0.02–0.10)
DHA C22:6	0.49 ± 0.05	0.5 ± 0.2	0.67 ± 0.47	0.18 ± 0.12	0.28 (0.17)	0.7 ± 0.3	0.62 (0.31–0.93)	0.13 (0.04–0.21)
Total n-6 PUFA	11.2 ± 0.42	24.1 ± 5.0	18.2 ± 3.9	14.80 ± 3.09	10.61 (3.17)	12.1 ± 2.7	12.47	20.58
Linoleic Acid C18:2	10.0 ± 0.40	22.8 ± 4.9		13.62 ± 3.01	9.35 (2.90)	11.1 ± 2.6	9.31 (3.86–14.8)	18.1 (12.8–24.1)
Arachidonic Acid C20:4	0.39 ± 30.03	0.7 ± 0.2	0.48 ± 0.13	0.38 ± 0.09	0.20 (0.04)	0.5 ± 0.1	0.96 (0.44–1.48)	0.56 (0.43–0.69)
n-6–n-3 ratio	10.1 ± 3.77	12.5 ± 5.5	6.7 ± 2.0	6.53 ± 1.72	9.58 (3.31)	4.6 ± 1.0	3.48	7.56

Malaysia: current study. China (cities Beijing, Suzhou, and Guangzhou): Guiffrida et al., 2016 [34]. South Korea: Kim et al., 2017 [7]. Canada: Moossavi et al., 2019 [35]. Switzerland: Thakkar, 2019 [36]. Poland: Bzikowska–Jura et al., 2019 [26]. * Bolivia and * USA: Innis, 2014 [6]; * Bolivia and * USA: Martin et al., 2012 [37].

## Data Availability

The data presented in this study are available within the article.
